# App Characteristics and Accuracy Metrics of Available Digital Biomarkers for Autism: Scoping Review

**DOI:** 10.2196/52377

**Published:** 2023-11-17

**Authors:** Sonia Ponzo, Merle May, Miren Tamayo-Elizalde, Kerri Bailey, Alanna J Shand, Ryan Bamford, Jan Multmeier, Ivan Griessel, Benedek Szulyovszky, William Blakey, Sophie Valentine, David Plans

**Affiliations:** 1 Healios Limited London United Kingdom; 2 Institute of Health Informatics University College London London United Kingdom; 3 Institute of Epidemiology and Healthcare University College London London United Kingdom; 4 School of Computing, Engineering and Physical Sciences University of the West of Scotland Paisley United Kingdom; 5 Department of Psychology Royal Holloway, University of London London United Kingdom

**Keywords:** autism, diagnostics, digital biomarkers, digital health, mobile apps, neurodevelopmental conditions

## Abstract

**Background:**

Diagnostic delays in autism are common, with the time to diagnosis being up to 3 years from the onset of symptoms. Such delays have a proven detrimental effect on individuals and families going through the process. Digital health products, such as mobile apps, can help close this gap due to their scalability and ease of access. Further, mobile apps offer the opportunity to make the diagnostic process faster and more accurate by providing additional and timely information to clinicians undergoing autism assessments.

**Objective:**

The aim of this scoping review was to synthesize the available evidence about digital biomarker tools to aid clinicians, researchers in the autism field, and end users in making decisions as to their adoption within clinical and research settings.

**Methods:**

We conducted a structured literature search on databases and search engines to identify peer-reviewed studies and regulatory submissions that describe app characteristics, validation study details, and accuracy and validity metrics of commercial and research digital biomarker apps aimed at aiding the diagnosis of autism.

**Results:**

We identified 4 studies evaluating 4 products: 1 commercial and 3 research apps. The accuracy of the identified apps varied between 28% and 80.6%. Sensitivity and specificity also varied, ranging from 51.6% to 81.6% and 18.5% to 80.5%, respectively. Positive predictive value ranged from 20.3% to 76.6%, and negative predictive value fluctuated between 48.7% and 97.4%. Further, we found a lack of details around participants’ demographics and, where these were reported, important imbalances in sex and ethnicity in the studies evaluating such products. Finally, evaluation methods as well as accuracy and validity metrics of available tools were not clearly reported in some cases and varied greatly across studies. Different comparators were also used, with some studies validating their tools against the Diagnostic and Statistical Manual of Mental Disorders criteria and others through self-reported measures. Further, while in most cases, 2 classes were used for algorithm validation purposes, 1 of the studies reported a third category (indeterminate). These discrepancies substantially impact the comparability and generalizability of the results, thus highlighting the need for standardized validation processes and the reporting of findings.

**Conclusions:**

Despite their popularity, systematic evaluations and syntheses of the current state of the art of digital health products are lacking. Standardized and transparent evaluations of digital health tools in diverse populations are needed to assess their real-world usability and validity, as well as help researchers, clinicians, and end users safely adopt novel tools within clinical and research practices.

## Introduction

Autism is a common form of neurodivergence estimated to affect 1% of the population worldwide, with prevalence rates reported at 1% in the United Kingdom [1] and 1.85% in the United States [2]. Despite this high prevalence rate, diagnostic delays are common, with difficulties receiving an initial service referral [1] and families in the United Kingdom [3] and United States [4] reporting between 2 and 3 years to receive a diagnosis from the onset of symptoms, respectively. On top of severely impacting the quality of life of those awaiting assessments and their families [5], diagnostic delays may increase the likelihood and severity of comorbidities [6]. Further, current diagnostic processes for autism rely solely on subjective clinician interpretations derived from standardized assessment tools, leading to potential misdiagnosis [7] and accentuation of phenomena such as masking [8].

Digital health products, such as mobile apps, have the potential to aid the diagnostic process due to their scalability and ease of access. Apps also offer the possibility of providing additional ecological information collected in users’ home environments to clinicians during the assessment phase. Specifically, digital biomarkers, that is, digital tools that collect information about the behavioral characteristics and physiological processes of individuals affected by a condition, have shown promise in identifying the presence of a disorder in several diagnostic domains (eg, cognitive impairment and dementia [9], depression [10], and learning disabilities [11]). Recommendations for developers and the scientific community at large outline the importance of transparent communication of the algorithms underlying digital biomarkers as well as plans for iterative evaluations of such products [12]. Further, multimodal approaches that prioritize cognitive and behavioral assessments have been recognized as promising in aiding precision diagnostics and personalized therapeutics [13]. While research on digital biomarkers in autism is still in its infancy and specific recommendations for the development and validation of these products are lacking, there are digital health tools available to researchers, clinicians, and end users.

Nevertheless, not many resources synthesizing the characteristics and evaluation outcomes of these products are widely available. The aim of the current scoping review is to summarize the evidence about existing digital biomarker tools so that researchers in the field of autism, clinicians, and end users are provided with up-to-date information to make informed decisions regarding their usefulness and adoption.

## Methods

### Search Strategy

A structured search, following the PRISMA-ScR (Preferred Reporting Items for Systematic Reviews and Meta-Analyses extension for Scoping Reviews) guideline [14], was conducted on August 21, 2023 (with further manual searches conducted ad hoc), in the databases MEDLINE through PubMed and Elsevier’s Scopus. The search terms included “autism digital biomarker”; “autism app”; and related synonyms, truncations, and Medical Subject Headings. Additional searches were conducted through the US Food and Drug Administration (FDA) website [15] and Google to find regulatory submissions and additional materials, respectively. If data reported as part of a regulatory submission had been published in a peer-reviewed journal, the peer-reviewed journal was used. A review protocol was not published; however, the full search strategy can be found in Table S1in Multimedia Appendix 1.

### Inclusion and Exclusion Criteria

Only full-text primary research studies published in English and in peer-reviewed journals, as well as regulatory submissions published on the regulatory body website, were included (Table S2 in Multimedia Appendix 1). Further, studies were included if they reported accuracy and validity metrics from available digital biomarkers. Apps using digital versions of existing standardized autism assessments or telehealth adaptations of in-person assessments were also excluded.

### Data Extraction and Analysis

Data were extracted and analyzed by 3 reviewers. Titles and abstracts were screened once with a reason for exclusion provided by 1 reviewer, which was then inspected by another reviewer. Full texts of eligible papers were reviewed independently by at least 2 reviewers, and the inclusion and exclusion of studies were discussed as a team. The full data extraction form can be found in Multimedia Appendix 1; Table S3 in Multimedia Appendix 1 provides further study details and metrics about the included studies.

## Results

### Overview

**Figure 1:**
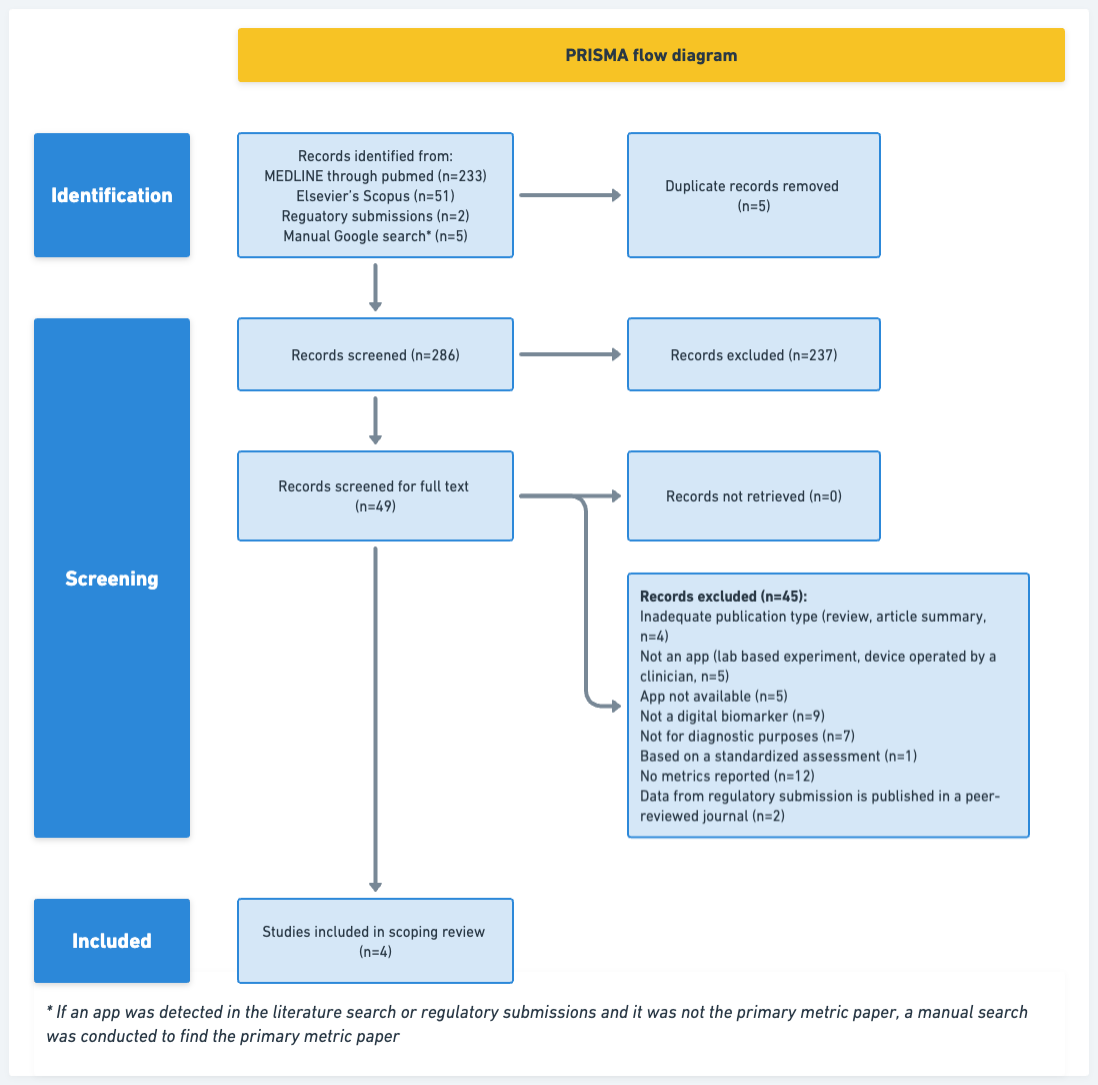
PRISMA (Preferred Reporting Items for Systematic Reviews and Meta-Analyses) flow diagram of the literature search and selection process.

We found 286 studies and regulatory submissions, of which 49 were eligible for full-text screening. A total of 4 studies met our criteria and were included in the review (Figure 1).

### App Characteristics and Regulatory Aspects

**Figure 2:**
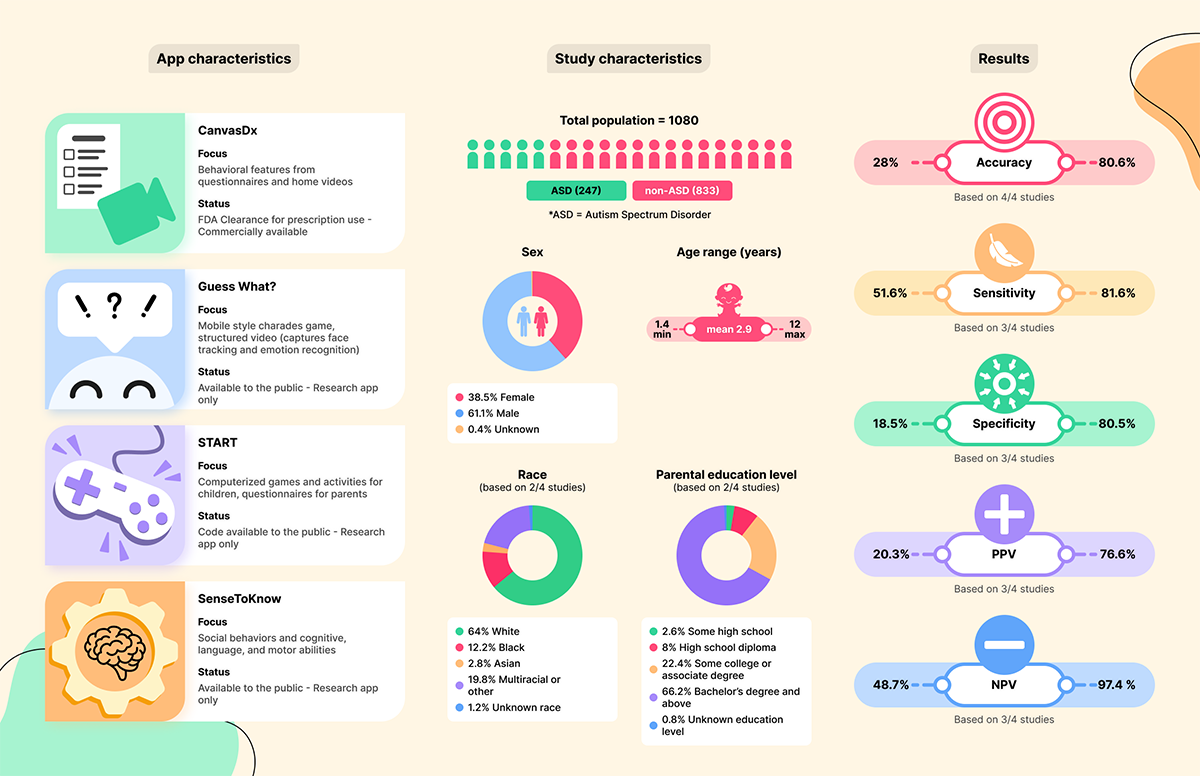
Summary of app characteristics, study details, and accuracy metrics for all included studies. ASD: autism spectrum disorder; FDA: US Food and Drug Administration; NPV: negative predictive value; PPV: positive predictive value.

A total of 4 studies involving 4 unique apps were included in the review (Figure 2), of which 1 (Canvas Dx [16]) was an FDA-cleared commercial product and 3 (Guess What? [17], START [18], and SenseToKnow [19]) were research apps.

The apps from the included studies target children between 17 and 144 months (1.4-12 years) of age who do not exhibit significant sensory or motor impairments (due to the nature of the tasks). Canvas Dx uses questionnaires from caregivers and health practitioners and videos to provide a diagnostic indication. Guess What? acquires structured videos of the interaction between child and parent during a charades-style game and applies face tracking and emotion recognition techniques. START measures social, sensory, and motor skills through games and activities for children and a questionnaire for parents. SenseToKnow displays specifically designed movies aimed at eliciting autism-related attention and motor behaviors while recording the child’s responses through the front camera of the device.

As for their technical approaches, 2 studies were administered through smartphones and 2 through tablets. Most apps (3/4, 75%) classified individuals as either autistic or nonautistic, although CanvasDx included an “undetermined” category. Further, all the studies used tree-based classification methods, particularly gradient-boosted decision trees. In terms of real-world usability, most studies (3/4, 75%) reported results from usability testing, interviews with clinicians and families, or app quality scores, showing good acceptability and feasibility results and high (93.9%) quality scores. All tools emphasize that their intended use is screening or diagnostic aid rather than stand-alone diagnostics. CanvasDx also warns that results may be potentially unreliable in individuals with specific medical conditions, such as epilepsy or genetic disorders (Table S4 in Multimedia Appendix 1).

A total of 3 out of the 4 apps from the included studies are available for download in the United States (links are included in Table S3 in Multimedia Appendix 1), with 1 (Guess What?) also being available in the United Kingdom. As for START, only the app code has been published. All apps are free to download. Regarding device compatibility, Canvas Dx and Guess What? can be downloaded on either Android or iOS; SenseToKnow is only available for iOS; and the code for START is for Android implementation only.

### Studies Characteristics

Studies included to validate these 4 apps involved 1080 individuals. Participants were children aged between 17 and 144 months (1.4-12 years; mean 2.9 SD 1.0 years), with a mean autism prevalence of 22.9% (range 10.3%-57.1%). The pooled sex split was 38.5% (416/1080; range 30.5%-43.4%) female, 61.1% (660/1080; range 56.6%-69.5%) male, and 0.4% (4/1080; 1 study only: 8.2%) unknown. Race was reported for 2 studies: 2.8% (25/900; range 1.5%-4.2%) Asian, 12.2% (110/900; range 11.4%-13.2%) Black, 64% (576/900; range 53.9%-73.2%) White, 19.8% (178/900; range 13.9%-28.7%) multiracial or other, and 1.2% (11/900; range 0.2%-2.4%) unknown, and ethnicity only reported independently in 1 study: 10.5% (50/475) Hispanic or Latino and 89.5% (425/475) non-Hispanic or non-Latino. A total of 2 studies also reported parental education level: 2.6% (23/900; range 2.1%-3.1%) some high school, 8% (72/900; range 5.9%-10.4%) high school diploma, 22.4% (202/900; range 11.2%-35.1%) some college or associate degree, 66.2% (596/900; range 50.4%-80.4%) bachelor’s degree and above, and 0.8% (7/900; range 0.4%-1.2%) unknown. Samples varied in size and ranged from 49 to 475.

### Accuracy and Validity Metrics

When metrics were not reported by the authors but sensitivity, specificity, and prevalence were indicated, they were calculated using the formulas in Table 1. Overall, accuracy fluctuated between 28% and 80.6%. Sensitivity and specificity also varied, ranging from 51.6% to 81.6% and 18.5% to 80.5%, respectively. Similarly, positive predictive value ranged from 20.3% to 76.6%, and negative predictive value fluctuated between 48.7% and 97.4%.

**Table 1:** Accuracy and validity metrics of the included digital biomarker apps for autism classification.

App name	Sample size, n	Autism prevalence (%)	Accuracy (%)	Sensitivity (%)	Specificity (%)	PPV^a^ (%)	NPV^b^ (%)	Comparator
Canvas Dx	425	28.7	28^c^	51.6^d^	18.5^d^	20.3^c^	48.7^c^	ECD^e^
Guess What?^f^	49	57.1	73	76	69^c^	76.6^c^	68.3^c^	Parent-reported diagnosis
START	131	36.6	61.6	Unable to calculate	Unable to calculate	Unable to calculate	Unable to calculate	ECD
SenseToKnow	475	10.3	80.6^c^	81.6	80.5	32.5^c^	97.4^c^	ECD

^a^PPV: positive predictive value.

^b^NPV: negative predictive value.

^c^Calculated using the following formulas: accuracy = (sensitivity) × (prevalence) + (specificity) × (1 – prevalence); PPV = (sensitivity × prevalence) / {(sensitivity × prevalence) + ([1 – specificity] × [1 – prevalence])}; and NPV = (specificity × [1 – prevalence]) / {(specificity × [1 – prevalence]) + ([1 – sensitivity] × prevalence)}.

^d^For 3 classes (autism, uncertain, and nonautism) classification. If participants who received an uncertain or indeterminate class decided by the classifier were removed, for the remaining 31.8% (135/425) of participants who received a determinate output (autism or nonautism), sensitivity and specificity increased to 98.4% and 78.9%, respectively.

^e^Expert clinician diagnosis.

^f^Metrics from the feasibility study. Ongoing study for the validation of the GuessWhat? app (registered trial NCT04739982 [20])

## Discussion

### Overview

This study investigated the available digital biomarker tools for autism diagnosis. We found 4 products targeted at children, exploring a variety of domains, ranging from attention and looking behaviors to analysis of social, sensory, and motor skills. Of the examined research studies, 1 included commercial apps with medical device classification, and 3 involved unregulated research apps.

All studies use digital biomarkers of known domains that have been shown to be indicative of autism [21]. Most products use video analysis (often accompanied by questionnaires) to extract features of interest, whereas 1 of the included tools collects behavioral measures from interactive tasks. Both parent questionnaires and batteries assessing children during interactive tasks are part of well-known diagnostic assessments for autism, for example, Autism Diagnostic Observation Schedule (ADOS-2) [22,23], Autism Diagnostic Interview-Revised [24], and TELE-ASD-PEDS [25]. While assessment of different domains ensures higher coverage of potentially relevant behaviors, the variety in the evaluated domains limits the comparability between tools. Nevertheless, each of these digital biomarkers has strengths and weaknesses. CanvasDx obtained FDA approval for commercialization and is therefore undergoing robust validation processes. Further, its classification algorithm is very sensitive to extreme cases (very high or very low risk of autism); however, the inclusion of an indeterminate class makes it more difficult to identify less severe cases. Another limitation of CanvasDx is that it is not clinician independent. The strengths of Guess What? include its blended diagnostic and therapeutic approach, which offers an end-to-end solution for users; its wider age range of use; and its availability in multiple geographies. Its diagnostic and therapeutic nature is also its biggest limitation, as this tool was not originally designed as a diagnostic tool, and evaluation data are limited. The START app combines multiple assessment domains (social functioning and motor and sensory behaviors) and has been validated with diverse communities in mind. Nevertheless, the validation study does not report information about misdiagnoses, which limits the interpretability of its results. Finally, SenseToKnow offers combined biomarkers for social behavior as well as cognitive, language, and motor abilities within an assessment shorter than 10 minutes. The major limitation in its validation data pertains to the lower prevalence of autism-positive cases, which highlights the need for further evaluations of the tool.

Availability to download the apps was restricted to specific geographies, with unclear documentation about accessibility. Further, 1 of the tools (START) has only been published within a code repository, thus limiting accessibility to the wider population. Given that one of the main advantages of mobile apps is their scalability and ease of access [26], restrictions based on geography may negatively impact the adoption of such products in both research and clinical settings.

In terms of study details, only 2 studies reported full demographic information and socioeconomic variables, despite research showing differences in the adoption of health apps highly correlate with higher education and income [27]. The fact that most parents reported having a bachelor’s degree or above further impacts the generalizability of the results to individuals from different socioeconomic backgrounds and poses questions around overall real-world usability. Both sex and race and ethnicity data were unbalanced, with most study participants being male and White. Evidence suggests that phenomena such as masking are more common in female individuals [28,29], thus highlighting the need for digital biomarkers to be validated in balanced sex populations. Similarly, findings outline longer diagnostic delays among minorities and underserved communities, with an associated lack of early interventions during pivotal developmental years [30,31]. Thus, if app developers fail to assess the usability, acceptability, and validity of these tools in underserved communities and diverse populations, the potential of digital health products to address barriers to entry to diagnostic and therapeutic pathways may be significantly reduced. As such, research evaluating digital biomarkers for autism should aim to assess their performance in these demographics to successfully increase health equity and help tackle diagnostic complexities.

Contrary to the Standards for Reporting of Diagnostic Accuracy Studies [32], half (2/4, 50%) of the resources did not provide the full array of accuracy and validity metrics nor the confusion matrix (Table S3 in Multimedia Appendix 1), with 1 of the studies (START) only reporting accuracy (but no sensitivity or specificity nor confusion matrix), which substantially limits the interpretability of their findings. The observed high variability in the presented app metrics may be justified by discrepancies in the evaluation methods. First, differences in the prevalence of autism substantially impact the calculation of classic accuracy metrics, therefore not rendering a reliable picture of the true classifiers’ performance and limiting generalizability [33]. Similarly, most studies (3/4, 75%) used 2 classes (autism vs nonautism) for their algorithm validation, whereas 1 added an “indeterminate” class. Additionally, the studies evaluated their apps using different comparators, with most (3/4, 75%) measuring the performance of their algorithms against expert clinician diagnosis based on the Diagnostic and Statistical Manual of Mental Disorders-5 (DSM-5) criteria and another 1 referring to parent-reported diagnosis. Notably, clinicians may reach a diagnostic outcome using different diagnostic criteria (eg, DSM-5 or International Classification of Diseases-11) or supplement their clinical judgment with the use of assessment tools (eg, ADOS-2) [34,35]. Additionally, geographic differences exist in parent-reported symptomology when using common assessment tools [36], of which accuracy and validity metrics have also been shown to vary by country [37]. The abovementioned factors significantly impact any attempt to draw direct comparisons between studies. As such, app developers and researchers should aim to validate their products within the context of standardized evaluation frameworks that advocate for validation in diverse populations and with multiple comparators. Further, findings should be reported in a uniform fashion and should include the prevalence of the disorder, full metrics, confusion matrices, binary classification, and comparator of choice to facilitate the assessment of the potential for digital biomarkers to represent effective screening tools for autism.

This review highlighted important gaps in the literature surrounding the development and testing of digital biomarkers for autism. First, it remains unclear whether and to what extent these tools are typically developed with input from both end users and clinicians [38]. Frameworks outlining guidelines for the design and development of digital biomarkers would help unify approaches across companies and research entities and ensure standards of quality and safety [39]. Our work also outlined exciting trends toward the development of mobile-first digital technologies. It has been shown that while individuals in underserved communities often do not have access to desktop computers or laptops, the availability of mobile phones is typically higher [18]. Therefore, developing mobile-first tools could help ensure health equity in communities where conditions like autism suffer from greater diagnostic delays [40]. Finally, digital biomarker tools typically provide ecologically valid information that may not otherwise be available to clinicians. As such, including these tools as part of the waiting list for autism assessments could provide clinicians with valuable information ahead of the assessment, which in turn could help prioritize more severe cases.

### Conclusions

Digital health products are increasingly gaining popularity, yet systematic syntheses of the current state of the art are lacking. Our work highlighted how diversity in the development and evaluation of digital biomarkers aiding in the detection of autism may impact their real-world usability and adoption in communities where these tools may have the most positive impact. As such, standardized and transparent development and evaluation frameworks, recommending assessing the validity of digital biomarkers in diverse populations, are needed to guide researchers, clinicians, and end users in making informed decisions about whether to consider their adoption within research settings and clinical pathways.

## Appendix

Multimedia Appendix 1 Information on the included app's metrics and on the search strategy.

Multimedia Appendix 2 PRISMA-ScR checklist.

## Abbreviations

ADOS-2: Autism Diagnostic Observation Schedule
DSM-5: Diagnostic and Statistical Manual of Mental Disorders-5
FDA: US Food and Drug Administration
PRISMA-ScR: Preferred Reporting Items for Systematic Reviews and Meta-Analyses extension for Scoping Reviews
